# Metabolic alterations in dairy cows with subclinical ketosis after treatment with carboxymethyl chitosan‐loaded, reduced glutathione nanoparticles

**DOI:** 10.1111/jvim.15894

**Published:** 2020-09-23

**Authors:** Chang Zhao, Yunlong Bai, Shixin Fu, Ling Wu, Cheng Xia, Chuang Xu

**Affiliations:** ^1^ College of Animal Science and Veterinary Medicine, Heilongjiang Bayi Agricultural University Daqing China

**Keywords:** ^1^H‐NMR, dairy cow, metabolomics, rGSH chitosan nanoparticles, subclinical ketosis

## Abstract

**Background:**

Subclinical ketosis (SCK) causes economic losses in the dairy industry because it reduces the milk production and reproductive performance of cows.

**Hypothesis/Objectives:**

To evaluate whether carboxymethyl chitosan‐loaded reduced glutathione (CMC‐rGSH) nanoparticles can alleviate the incidence or degree of SCK in a herd.

**Animals:**

Holstein dairy cows 21 days postpartum (n = 15).

**Methods:**

The trial uses a prospective study. Five cows with serum β‐hydroxybutyric acid (BHBA) ≥1.20 mmol/L and aspartate aminotransferase (AST) <100 IU/L were assigned to group T1, 5 cows with BHBA ≥1.20 mmol/L and AST >100 IU/L to group T2, and 5 cows with BHBA <1.00 mmol/L and AST <100 IU/L to group C. Carboxymethyl chitosan‐loaded reduced glutathione (0.012 mg/kg body weight per cow) was administered to cows in T1 and T2 once daily via jugular vein for 6 days after diagnosis. Serum from all groups were collected 1 day before administration, then on days 1, 3, 5, 7, 10, and 15 after administration to determine the changes in biochemical index and ^1^H‐NMR.

**Results:**

The difference in liver function or energy metabolism indices in T1, T2, and C disappeared at day 7 and day 10 after the administration (*P* > .05). Valine, lactate, alanine, lysine, creatinine, glucose, tyrosine, phenylalanine, formate, and oxalacetic acid levels, and decrease in isoleucine, leucine, proline, acetate, trimethylamine N‐oxide, glycine, and BHBA levels were greater (*P* < .05) at day 7 than day 0 for cows in T2.

**Conclusions and Clinical Importance:**

Carboxymethyl chitosan‐loaded reduced glutathione treatment might alleviate SCK by enhancing gluconeogenesis and reducing ketogenesis in amino acids.

**Abbreviations**
ALT: alanine aminotransferase
AST: aspartate aminotransferase
ALB: albumin
BHBA: β‐hydroxybutyric acid
BCS: body condition score
CMC: carboxymethyl chitosan
GLu: glucose
^1^H‐NMR: nuclear magnetic resonance
HDL‐C: high‐density lipoprotein
LDL‐V: low‐density lipoprotein
NEFA: free fatty acid
PCA: principal component analysis
PLS‐DA: partial least squares discrimination
rGSH: reduced glutathione
SCK: subclinical ketosis
TP: total protein
TC: total cholesterol
TG: triglyceride

## 1 INTRODUCTION

Subclinical ketosis (SCK) is 1 of the most common metabolic diseases,^1^ with an incidence of usually 15% to 30%, but more than 50% on some intensive cattle farms.^2^ High producing cows are susceptible to negative energy balance (NEB) because of the high lactational demand and insufficient dry matter intake (DMI) after calving, and NEB will stimulate the mobilization of body fat and protein to satisfy the nutrient demand of the lactating cow.^3^, ^4^ High levels of ketogenic amino acids (AAs) and nonesterified fatty acids (NEFA) are produced by the mobilization of fat and protein, resulting in ketosis because of the incomplete oxidation of ketone bodies.^5^, ^6^ Subclinical ketosis can reduce milk production and reproductive performance, and increase the incidence of other diseases, such as metritis, mastitis, and milk fever.^7^ Subclinical ketosis therefore causes huge economic loss in the dairy industry. Drugs used currently to treat simple ketosis include glucose (GLu),^8^ propylene glycol,^9^ and glycerin.^10^ These drugs have played an important role in the treatment of ketosis either because of the direct supplementation of GLu or the stimulation of gluconeogenesis.^11^, ^12^ However, it is difficult to clinically treat secondary ketosis with fatty liver or abnormal liver function in dairy cows resulting from a high amount of NEFA and lipid oxidative stress.^13^, ^14^ Subclinical ketosis is often overlooked or not treated effectively because of lack of signs. The challenge of exploring effective prophylactic preparations to alleviate SCK with concurrent fatty liver or abnormal liver function still exists.

The reduced glutathione (rGSH) participate in carbohydrate metabolism and the tricarboxylic acid cycle in vivo.^15^ In human and mouse metabolic syndrome and nonalcoholic fatty liver disease, rGSH is used as therapeutic and adjuvant drug that can reduce the production of free radicals, protecting the liver from injury by oxygen‐free radicals.^16^, ^17^, ^18^, ^19^ Studies have confirmed that cows with SCK have different degrees of oxidative stress and abnormal liver function indices.^20^, ^21^ However, there have not been any previous reports where carboxymethyl chitosan‐loaded reduced glutathione (CMC‐rGSH) nanoparticles have been used to treat or improve SCK in dairy cows. We propose that rGSH might improve SCK, with and without fatty liver, in dairy cows.

Metabolomics is a new branch of ‐omics.^22^ Nuclear magnetic resonance (^1^H‐NMR) is 1 of the metabolomics technologies, and has several advantages in simple sample pretreatment and it can accurately evaluate the efficacy of drugs.^23^ Plasma metabolic profiles of ketosis or SCK based on ^1^H‐NMR have been reported.^24^, ^25^, ^26^ It has become a strong tool/technology in the research of metabolic disease in dairy cattle.

In this study, ^1^H‐NMR was combined with multivariate statistical analysis to measure the effect of CMC‐rGSH nanoparticles on SCK‐affected cows. The purpose was to demonstrate that CMC‐rGSH nanoparticles can treat or alleviate SCK by improving AA, GLu, and lipid metabolism.

## 2 MATERIALS AND METHODS

### 2.1 Preparation of CMC‐rGSH nanoparticles

The specific production and quality evaluation of rGSH nanoparticles is reported by Zhu et al.^20^ Briefly, 4 g lactose and 15 mL potassium borohydride (KBH_4_ 0.1 g/mL) solution were added to 50 mL CMC‐loaded solution (10 mg/mL), and the mixture was stirred and centrifuged at 3000 rpm for 5 minutes. The supernatants were treated by dialysis to remove insoluble substances, and the filtrate was freeze dried to obtain the CMC. At room temperature, sodium alginate (0.2 mg/mL), calcium chloride (4 mg/mL), and CMC solution were added to a total of 50 mL solution (6 mg/mL) at a ratio of 5:2:6. The rGSH was added to the mixture and stirred to produce the required emulsion.

### 2.2 Animals and diet

The experiments were managed in accordance with the National Institute of Health Guidelines for the Nursing and Use of Laboratory Animals. All animals were managed in accordance with the standards approved by the Ethics Committee for Animal Welfare and Research of Heilongjiang Bayi Agricultural University China (Number: 20190321‐4). Holstein cows are bred in accordance with the conventional feeding and management standards for cows in China. The diet composition and nutrition level of dairy cows is shown in Table 1.

**TABLE 1:** Dietary composition and nutrition level of experimental cows

Diet composition (kg)		Nutrient content	
Water	5.47	CP (%)	17.70
Cottonseed	1.03	Starch (%)	22.70
Soybean hulls	1.50	DM (%)	48.00
Oat grass	0.50	NDF (%)	31.50
Alfalfa	2.50	ADF (%)	19.00
Soybean meal	1.30	FNDF (%)	18.70
Corn flakes	2.00	Net milk production (MCal/kg)	0.78
Molasses	1.00		
Silage	25.37		
corn	3.00		
Premix of minerals and trace elements	1.20		
Concentrated feed	4.09		
Total	48.96		

Abbreviations: ADF, acid detergent fiber; CP, crude protein; DM, dry matter; FNDF, source of roughage neutral detergent fiber NDF; NDF, neutral detergent fiber.

In this experiment, 15 dairy cows at 21 days *postpartum* were randomly assigned to 3 groups (T1, T2, and C) according to the individual plasma concentrations of β‐hydroxybutyric acid (BHBA) and aspartate aminotransferase (AST).^27^, ^28^, ^29^, ^30^, ^31^ These cows had a similar parity of 1.67 ± 0.13 fetuses, daily milk yield of 30.54 ± 2.71 kg/d, and a similar body condition score (BCS) of 4.0 ± 0.5 points. Among them, 5 cows with a plasma BHBA concentration ≥1.20 mmol/L and AST activity <100 IU/L were assigned to T1 group as SCK, 5 cows with 2.00 mmol/L ≥ BHBA concentration ≥ 1.20 mmol/L and AST activity >100 IU/L to T2 group as SCK, and 5 normal cows with BHBA <1.00 mmol/L and AST <100 IU/L were assigned to the control group C. Carboxymethyl chitosan‐loaded reduced glutathione nanoparticles (0.012 mg/kg body weight [BW] per cows) were administered to cows in T1 and T2 once daily (by jugular vein, IV.) for 6 days after diagnosing SCK. The dosage, and days of treatment with CMC‐rGSH of SCK cows was speculated based on the previous pharmacodynamic studies reported by Zhu et al.^20^, ^21^, ^32^ There were no other clinical abnormalities or diseases in the 3 groups of cows during the experiment. At the same time, the DMI, parity, and BCS of the dairy cows in each group were recorded for 21 days after treatment.

### 2.3 Sample collection

For assessing the effect of CMC‐rGSH nanoparticles on SCK before and after diagnosis, blood samples from the 3 groups (T1, T2, and C) were taken at 0600 hours, before the morning feeding, from the tail vein on day 1 before administration of CMC‐rGSH nanoparticles, and then on days 1, 3, 5, 7, 10, and 15 after administration. The method of Zhu et al^20^, ^21^, ^32^ refers to the effect of CMC‐rGSH in rat with fatty liver syndrome. All collected blood samples were divided into 2 parts. One part was added into a heparin tube and centrifuged at 3500 rpm for 10 minutes to separate the plasma, which was the stored at −20°C for biochemical analysis. The rest was allowed to coagulate, and then centrifuged at 3500 rpm for 10 minutes to separate the plasma, which was stored at −80°C for ^1^H‐NMR analysis.

### 2.4 Plasma biochemical indicator analysis

Plasma biochemical index include indicators for energy metabolism, liver function, and lipid metabolism. The energy metabolism indicators include BHBA, NEFA, and GLu (mmol/L); liver function indicators include total AST (IU/L), alanine aminotransferase (ALT IU/L), protein (TP g/L), albumin (ALB g/L), and globulin (g/L); lipid metabolism indicators include total cholesterol (TC mmol/L), triglyceride (TG mmol/L), high‐density lipoprotein (HDL mmol/L), and low‐density lipoprotein (LDL mmol/L). The presence of SCK was determined by testing plasma BHBA using a blood ketone meter and ketosis reagent strips with 93.8% sensitivity, 100% specificity, and a 93.8% Youden index (Yicheng, Beijing, China). Other biochemical parameters were measured using commercial kits and instruments from our previous research.^33^ Statistical analysis was carried out using SPSS 19.0 (SPSS, IBM, Armonk, New York).

### 2.5 Plasma ^1^H‐NMR analysis

^1^H‐NMR was performed on a 500 MHz ^1^H‐NMR spectrometer (AVANCE III, Bruker, Switzerland). A serum sample (400 μL) was mixed with 600 μL of 99.8% D_2_O phosphate buffer solution (0.2 mol/L Na_2_HPO_4_, 0.2 mol/L NaH_2_PO_4_, pH = 7.0) containing 0.05% (w/v) TSP, and was centrifuged at 12 000 rpm × 10 minutes, and the supernatant was taken. The test temperature was 298 K, and the pulse sequence used was the Carr‐Purcell‐Meiboom‐Gill (CPMG) sequence (RD‐90 [τ‐180‐τ] n‐ACQ), with a total spin echo delay (2nτ) of 10 ms. The number of ^1^H‐NMR acquisition scans (NS) was 32, the number of sampling points (TD) was 32 k, and the spectral width was 10 000 Hz. All ^1^H‐NMR data needed to be corrected for zero‐point, phase, and baseline in Topspin software (Bruker, Germany). The sample spectrum was integrated at 0.015 ppm in the interval of 0.8 to 8.5 ppm, chemical shift, the water peak, and its influence area were removed from the resonance signal in the range of 4.5 to 5.18 ppm.

All spectrum data were normalized by probabilistic quotient normalization (PQN), and then normalized by Pareto‐scale and mean‐center functions. Identification of compounds was done using Chenomx software (Chenomx, Canada) to fit and compare the peaks, select compounds with good peak shape matches, and combine the metabolomics databases Madison metabolomics consortium database and human metabolome database. These were then analyzed with Statistical Total Correlation spectroscopy. Method auxiliary identification of peak metabolites was performed on ^1^H‐NMR spectra. All integrated data were normalized and integrated to perform multivariate statistical analysis as described by Zhang et al.^34^

### 2.6 Multivariate statistical analysis

SIMCA‐P10.0 (Sweden, Umetrics AB, Umea, Sweden) was used to perform multivariate statistical analysis on the data, including principal component analysis (PCA), partial least squares discrimination (PLS‐DA), and orthogonal signal correction (OPLS‐DA). Principal component analysis of plasma metabolite ^1^H‐NMR signals, analysis, and comparison of the overall presentation of sample distributions, judgment of differences between groups, and determination of the principal components was conducted. Partial least squares discrimination uses a 2‐fold, cross‐validation method to test the quality of the model and uses R^2^X and Q^2^ (representing the model's interpretable variables, and the model's predictability, respectively) to evaluate the model's effectiveness. Through OPLS‐DA analysis for the correlation coefficient of each metabolite, statistically significant metabolites were further summarized. Orthogonal signal correction was obtained after orthogonal signal correction on the PLS‐DA model. Orthogonal signal correction can remove variables that have no meaning for grouping, thereby maximizing the difference between groups.

Combined with a 1‐way analysis of variance (ANOVA), this experiment set 0.05 as the threshold for screening differentially expressed metabolites. Differential metabolites causing differences between groups were obtained. The KEGG (https://www.kegg.jp) database was used to search the metabolic pathways related to the differential metabolites.

## 3 RESULTS

### 3.1 Effect of GCMC‐rGSH nanoparticles on energy metabolism, liver function and lipid metabolism in SCK dairy cows

**FIGURE 1:**
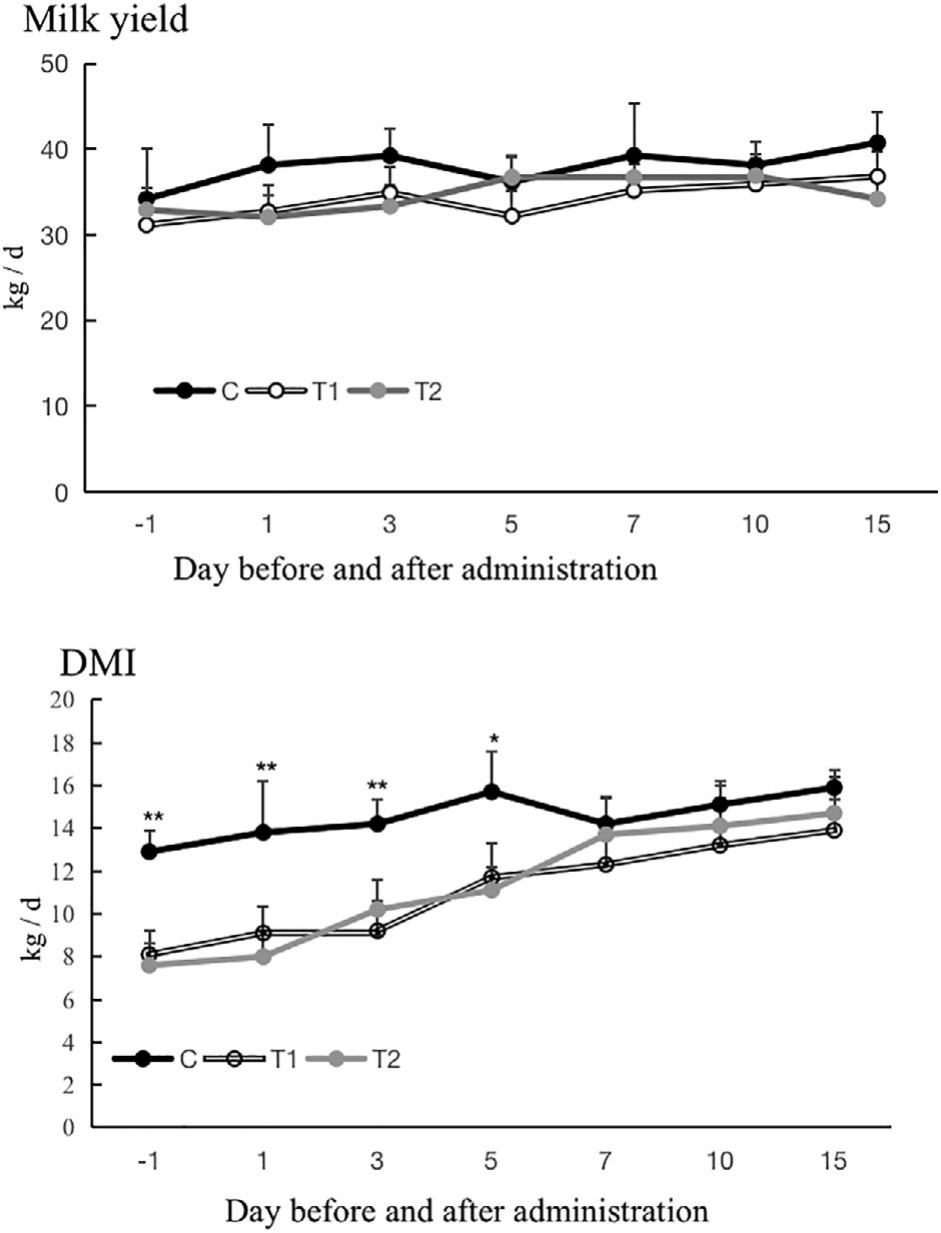
Dry matter intake (DMI) and milk yield after administration. ** represents a significant difference between groups (*P* < .01) and * represents a significant difference between groups within the same time period (*P* < .05)

Figure 1 shows the levels and changes in DMI and milk yield of *postpartum* dairy cows in the T1, T2, and C groups. On day 1 before administration, and days 1, 3, and 5 after administration, the DMI in group C was higher than T1 and T2 (*P* < .01). On days 7, 10, and 15 after administration, there were no differences in DMI between group C and groups T1 and T2 (*P* > .05). The DMI in the T1 and T2 groups showed an increase after treatment. There was a slight increase in milk yield in the 3 groups (C, T1, and T2) over the experiment, but milk yield in group C was higher than group T1, T2 (*P* > .05) the whole period. Table 2 shows the energy metabolism, liver function, and lipid metabolism indices of the 3 groups of experimental cows on different days after administration of CMC‐rGSH. Day 1 (before administration), the levels of energy metabolism indicators (BHBA, NEFA), and liver function indicators (ALT, AST, TG, ALB) in the plasma of the cows in T1 and T2 were higher than those in the C group (*P* < .01). Lipid metabolic indicators (TC, HAL‐C, LDL) and GLu in the plasma of T1 and T2 were lower than in group C (*P* < .01). After administration, the serum levels of ALT, AST, TG, NEFA, and BHBA in T1 and T2 cows decreased, and the levels of TC, HDL, LDL, and GLu increased. Compared with group C, there were differences in the ALT, AST, TC, TG, HDL, LDL, NEFA, BHBA, and GLu in the serum of the T1 and T2 groups before administration. Five days after administration, TC and HDL differences in the serum of the T, T2, and C groups disappeared. Seven days after administration, the differences in AST, TG, LDL, BHBA, and GLu in the serum of T1, T2, and C groups disappeared. Ten days after administration, the difference in NEFA in the serum of groups T1, T2, and C disappeared. On day 15 after administration, the difference in ALT in the serum of T2 and C disappeared.

**TABLE 2:** Plasma biochemical index of dairy cows with subclinical ketosis (SCK) before and after administration of CMC‐rGSH (mean ± SD, n = 5)

Item	Group	Day before and after administration (day)						
		−1	1	3	5	7	10	15
ALT	T1	39.33 ± 3.57^A^	37.33 ± 8.01^A^	35.33 ± 3.60^A^	32.06 ± 2.08^Aa^	29.33 ± 4.16^A^	30.01 ± 3.13^a^	27.00 ± 1.39
	T2	45.68 ± 4.26^B^	44.92 ± 9.39^B^	42.17 ± 8.50^B^	41.33 ± 3.05^B^	39.37 ± 0.57^B^	34.00 ± 2.28^b^	31.68 ± 5.50
	C	26.62 ± 8.62^C^	27.56 ± 1.87^C^	26.81 ± 3.11^C^	29.21 ± 4.64^Ab^	29.45 ± 6.54^A^	28.20 ± 1.32^a^	29.66 ± 5.50
AST	T1	109.33 ± 10.52^A^	106.41 ± 12.53^A^	101.33 ± 13.51^Aa^	100.28 ± 13.60^a^	85.33 ± 7.76	85.00 ± 9.10	85.33 ± 8.13
	T2	137.33 ± 9.52^B^	123.63 ± 13.45^B^	121.33 ± 16.50^Ab^	109.71 ± 9.46^a^	89.33 ± 9.51	88.03 ± 6.21	87.33 ± 10.88
	C	89.25 ± 6.34^C^	88.28 ± 8.38^C^	87.64 ± 14.66^C^	88.24 ± 4.14^b^	87.20 ± 10.70	85.60 ± 9.03	88.66 ± 9.16
TP	T1	69.06 ± 5.39	71.76 ± 1.30	67.73 ± 3.47	68.50 ± 4.82	70.03 ± 1.81	70.86 ± 4.17	77.10 ± 2.56
	T2	70.63 ± 4.55	69.96 ± 1.30	70.53 ± 2.75	77.60 ± 9.45	78.13 ± 7.41	71.13 ± 1.10	73.86 ± 6.62
	C	71.98 ± 3.47	70.02 ± 7.02	70.41 ± 2.53	72.14 ± 4.22	72.47 ± 2.40	74.55 ± 5.45	71.22 ± 4.23
ALB	T1	56.26 ± 7.40	55.67 ± 1.93	56.46 ± 1.70	55.93 ± 3.38	54.00 ± 2.51	49.03 ± 2.99	42.26 ± 6.96
	T2	54.41 ± 1.45	56.33 ± 1.30	56.06 ± 1.35	54.93 ± 6.35	52.86 ± 1.51	47.50 ± 2.51	41.50 ± 9.25
	C	50.78 ± 2.79	49.72 ± 4.55	48.08 ± 6.82	48.16 ± 8.28	48.51 ± 2.19	48.86 ± 1.62	47.46 ± 2.50
GLO	T1	23.83 ± 2.51	25.76 ± 2.04	21.26 ± 3.13	22.56 ± 2.41	26.03 ± 1.91	26.83 ± 7.08	26.83 ± 9.31
	T2	24.23 ± 6.00	22.63 ± 5.41	24.46 ± 1.65	22.66 ± 4.27	24.26 ± 4.94	23.63 ± 0.41	22.36 ± 6.41
	C	22.20 ± 1.85	25.29 ± 2.15	22.33 ± 2.17	23.97 ± 3.69	24.95 ± 2.33	25.70 ± 4.23	23.73 ± 2.40
TC	T1	3.54 ± 2.21^A^	3.39 ± 0.79^A^	4.59 ± 0.81^a^	4.83 ± 1.48	4.82 ± 1.64	4.75 ± 0.60	4.79 ± 1.06
	T2	3.25 ± 0.85^A^	3.13 ± 0.58^A^	4.13 ± 0.92^b^	5.01 ± 0.15	4.80 ± 1.64	4.73 ± 1.23	4.53 ± 2.33
	C	5.08 ± 0.43^B^	5.12 ± 0.78^B^	5.03 ± 1.52^c^	4.97 ± 0.52	4.99 ± 0.79	4.85 ± 1.85	4.75 ± 0.70
TG	T1	0.58 ± 0.04^Aa^	0.59 ± 0.03^A^	0.40 ± 0.05^Aa^	0.39 ± 0.06^a^	0.32 ± 0.06	0.33 ± 0.04	0.32 ± 0.08
	T2	0.64 ± 0.09^Ab^	0.78 ± 0.07^B^	0.54 ± 0.04^B^	0.42 ± 0.08^b^	0.31 ± 0.01	0.26 ± 0.02	0.29 ± 0.04
	C	0.31 ± 0.02^B^	0.36 ± 0.03^C^	0.31 ± 0.06^Ab^	0.32 ± 0.04^c^	0.28 ± 0.09	0.29 ± 0.02	0.30 ± 0.03
HDL	T1	1.92 ± 0.39^Aa^	1.96 ± 0.39^A^	2.29 ± 0.26^a^	2.33 ± 0.64	2.35 ± 0.03	2.42 ± 0.25	2.39 ± 0.37
	T2	1.84 ± 0.40^Ab^	1.88 ± 0.11^A^	2.18 ± 0.10^a^	2.21 ± 0.23	2.44 ± 0.17	2.40 ± 0.84	2.35 ± 0.39
	C	2.53 ± 0.37^B^	2.53 ± 0.33^B^	2.40 ± 0.17^b^	2.28 ± 0.18	2.44 ± 0.10	2.50 ± 0.71	2.32 ± 0.23
LDL	T1	1.82 ± 0.41^Aa^	1.88 ± 0.49^Aa^	2.17 ± 0.73^a^	2.04 ± 0.22^a^	2.89 ± 0.47	2.80 ± 0.74	2.76 ± 0.92
	T2	1.57 ± 0.25^Ab^	1.50 ± 0.65^Ab^	2.09 ± 0.88^a^	2.51 ± 0.38^b^	2.88 ± 0.33	2.82 ± 0.73	2.69 ± 0.15
	C	2.84 ± 0.18^B^	2.88 ± 0.62^B^	2.73 ± 0.46^b^	2.62 ± 0.61^b^	2.80 ± 0.82	2.80 ± 0.65	2.83 ± 0.27
NEFA	T1	0.79 ± 0.12^A^	0.81 ± 0.15^A^	0.69 ± 0.05^A^	0.45 ± 0.18^Aa^	0.51 ± 0.42^a^	0.40 ± 0.65	0.38 ± 0.45
	T2	0.86 ± 0.13^A^	0.87 ± 0.02^A^	0.73 ± 0.29^A^	0.66 ± 0.10^B^	0.52 ± 0.17^a^	0.45 ± 0.11	0.31 ± 0.34
	C	0.32 ± 0.14^B^	0.39 ± 0.20^B^	0.39 ± 0.27^B^	0.35 ± 0.15^Ab^	0.39 ± 0.18^b^	0.39 ± 0.36	0.31 ± 0.43
BHBA	T1	1.66 ± 0.24^A^	1.64 ± 0.61^A^	1.43 ± 0.23^A^	1.06 ± 0.29^Aa^	0.94 ± 0.11	0.99 ± 0.65	0.92 ± 0.19
	T2	1.72 ± 0.19^A^	1.60 ± 0.83^A^	1.51 ± 0.62^A^	1.25 ± 0.17^B^	1.06 ± 0.14	1.04 ± 0.23	0.99 ± 0.26
	C	0.86 ± 0.12^B^	0.61 ± 0.40^B^	0.90 ± 0.17^B^	0.79 ± 0.31^Ab^	0.89 ± 0.26	0.76 ± 0.39	0.73 ± 0.18
Glu	T1	3.02 ± 0.60^A^	3.79 ± 0.17^A^	3.60 ± 0.82^A^	3.81 ± 0.94^a^	4.39 ± 0.37	4.47 ± 0.37	4.33 ± 0.58
	T2	3.17 ± 0.68^A^	3.18 ± 0.97^A^	3.32 ± 0.64^A^	4.07 ± 0.75^a^	4.24 ± 0.99	4.23 ± 0.53	4.12 ± 0.66
	C	4.34 ± 0.49^B^	4.54 ± 1.27^B^	5.66 ± 1.62^B^	4.51 ± 1.15^b^	4.44 ± 0.57	4.42 ± 0.60	4.46 ± 0.73

*Note*: Between each indicator, different capital letters in same column represent significant differences (*P* < .01), different lowercase letters represent a significant difference (.01 < *P* < .05), and the same letter or without letters mean no significant difference (*P* > .05).

Abbreviations: Liver function indicators: ALB, albumin (g/L); ALT, alanine aminotransferase (U/L); AST, aspartate aminotransferase (U/L); GLO, globulin (g/L); TP, total protein (g/L). Lipid metabolism indicators: HDL, high‐density lipoprotein; LDL, low‐density lipoprotein; TC, total cholesterol (mmol/L); TG, triglyceride (mmol/L). Energy metabolism indicators: BHBA, β‐hydroxybutyric acid (mmol/L); GLu, glucose (mmol/L); NEFA, free fatty acid (mmol/L).

### 3.2 Plasma metabolite spectrum

**FIGURE 2:**
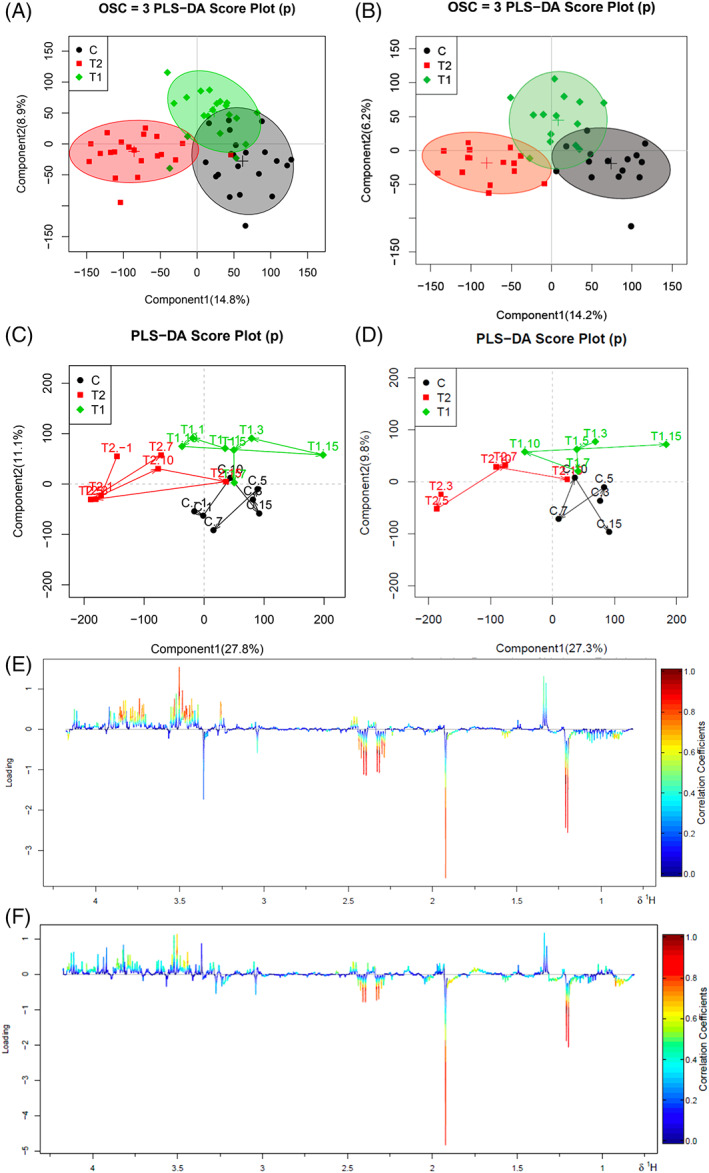
A, Partial least squares discrimination (PLS‐DA) score plot of plasma from 3 groups at all time points. B, Partial least squares discrimination score plot of plasma from 3 groups after administration. C, The time point trajectory of plasma from 3 groups at all time points. D, The time point trajectory of plasma from 3 groups at all time points after administration. E, Partial least squares discrimination loading plot of plasma from 3 groups at all time points. F, Partial least squares discrimination loading plot of plasma from 3 groups at all time points after administration. The darker the color, the greater the variation. Metabolites above the baseline were higher in the T1 group, and metabolites below the baseline were higher in the T2 group. Red dots represent group T2, green dots represent group T1, and black dots represent group C

The PLS‐DA score map of plasma metabolites of the 3 groups at all time points is shown in Figure 2A. Figure 2B is a plot of PLS‐DA scores at days 3, 5, 7, 10, and 15 after administration. There was a significant separation between the 3 groups. There is a 95% confidence interval between the samples in the circle. From Figure 2A,B, the separation between group C and T2 is better than between group C and T1.

Figure 2C shows the PLS‐DA trajectory analysis results of the 3 groups of serum samples before and after administration, and Figure 2D is the PLS‐DA trajectory analysis diagram of the 3 groups of serum samples at different time points after administration. Before administration, serum samples of the 3 groups of cows were separated. The differences between the 3 groups disappeared as the trial progressed. The metabolomics results were consistent with the serum biochemical index test results. Seven days after drug treatment, the difference among T2, T1, and C disappeared, and by day 15, T2 was already included in group C. The rGSH nanoparticles influence serum metabolites of SCK cows and SCK cows with a high AST.

Compared with the control, the metabolites in the serum of T1 and T2 cows showed changes in upregulation and downregulation, indicating that CMC‐rGSH caused significant changes in endogenous metabolites in T1 and T2 cows, before and after treatment (Figure 2E,F).

**FIGURE 3:**
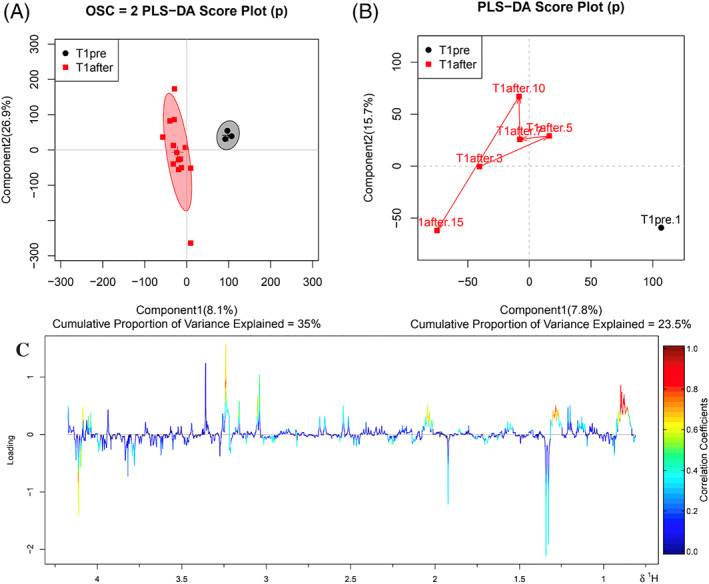
A. Partial least squares discrimination (PLS‐DA) scores at time points before and after plasma administration in the T1 group. B, Trace of PLS‐DA at time points before and after plasma administration in group T1. C, Partial least squares discrimination loading chart at time points before and after administration in group T1. The red dot represents after administration, and the black dot represents before administration

**FIGURE 4:**
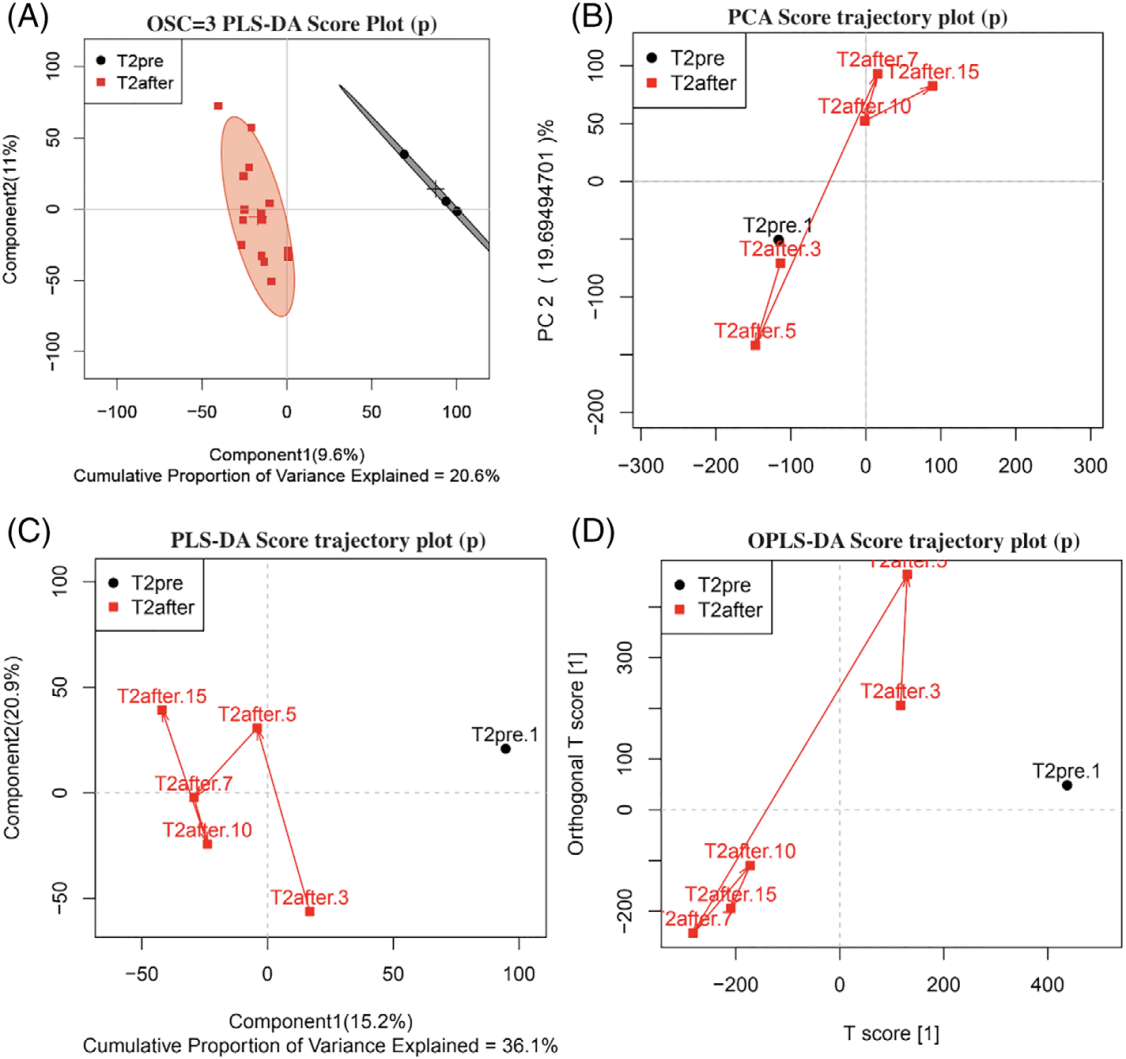
A, 3PLS‐DA (OSC) score chart before and after treatment in the T2 group. B, Principal component analysis score trajectory plot before and after treatment in the T2 group. C, Partial least squares discrimination score trajectory plot before and after treatment in the T2 group. D, Orthogonal signal correction score trajectory plot before and after treatment in the T2 group. The red represents after administration, black represents before administration

The sample separation before and after treatment was good, with differences between groups, as evidenced from the PLS‐DA score maps (Figures 3A and 4A). Carboxymethyl chitosan‐loaded reduced glutathione changed the metabolites in the serum of experimental cows. In the T1 group, the difference in plasma metabolites at 3, 5, 7, 10, and 15 days after administration did not change with the number of days after administration (Figures 3B and 4B). In the T2 group, PCA (Figure 4B), PLS‐DA (Figure 4C) trajectories, and OPLS‐DA (Figure 4D) trajectories all indicated that the metabolites in the serum of the T2 group varied with time after treatment. Serum metabolites showed differential changes among days 7, 10, and 15 after treatment, and 1 day before treatment, and days 3 and 5 after treatment. The rGSH nanoparticles were effective in cows with SCK and elevated AST, but not in cows with SCK without elevated AST.

**FIGURE 5:**
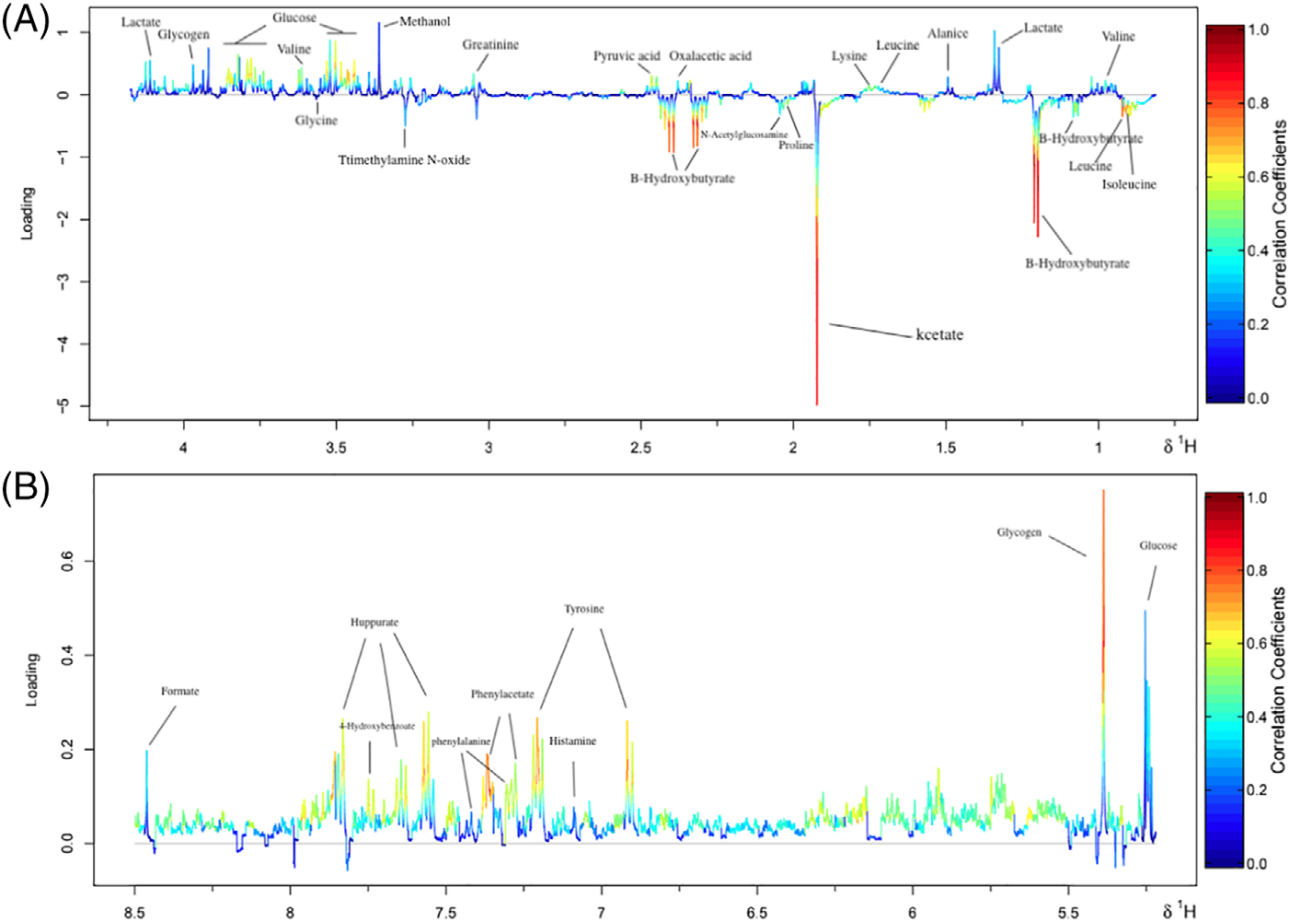
A, Partial least squares discrimination loading chart before and after administration in the T2 group (0‐5 ppm). B, Partial least squares discrimination loading chart before and after administration in the T2 group (5‐8.5 ppm). The differential metabolites in the serum of the T2 group before treatment and on the 7th day after treatment have been identified, and are marked in the figure

Because the results of the PLS‐DA analysis showed that CMC‐rGSH had a stronger therapeutic effect on T2 cows, with a more obvious difference in plasma metabolites, differential metabolites in the plasma of the T2 group before and after treatment were identified for bioinformatics analysis. Figure 5A (0‐5 ppm) and Figure 5B (5‐8.5 ppm) are loading graphs of the serum samples of the T2 group before treatment, and 7 days after treatment. Differential metabolites in the T2 group changed before and after treatment (Table 3). Valine, lactate, alanine, lysine, creatinine, GLu, tyrosine, phenylalanine, formate, and oxalacetic acid increased. Isoleucine, leucine, proline, acetate, trimethylamine N‐oxide, glycine, and BHBA decreased.

**TABLE 3:** The differential metabolites in the plasma of cows in group T2 before and on the 7th day after administration

No.	Metabolites	Chemical shift (ppm)	Changes after administration	*P*
1	Isoleucine	0.925‐0.955, 3.67‐3.69	↓	.05
2	Leucine	0.96‐0.98, 1.66‐1.73	↓	.02
3	Valine	0.98‐1.01, 1.04‐1.06, 3.61‐3.63	↑	.009
4	proline	1.01‐1.04	↓	.02
5	Lactate	1.32‐1.35, 4.09‐4.15	↑	.007
6	Alanine	1.475‐1.50	↑	.008
7	lysine	1.73‐1.77	↑	.008
8	Acetate	1.915‐1.93	↓	.01
9	Creatinine	3.045‐3.055, 4.055‐4.065	↑	.04
10	Glucose	3.39‐3.56, 3.70‐3.925, 5.23‐5.26	↑	.03
11	Trimethylamine N‐oxide	3.27‐3.28	↓	.01
12	Glycine	3.56‐3.57	↓	.04
13	Tyrosine	6.895‐6.925, 7.18‐7.21	↑	.03
14	Phenylalanine	7.315‐7.35, 7.41‐7.46	↑	.03
15	Formate	8.4‐8.55	↑	.02
16	BHBA	1.2, 2.31‐2.41	↓	.001
17	Oxalacetic acid	2.03‐2.055	↑	.05

*Notes*: ↓ represents a decrease in the differential metabolites after administration. ↑ represents an increase in the differential metabolites after administration.

### 3.3 Metabolic pathway

**FIGURE 6:**
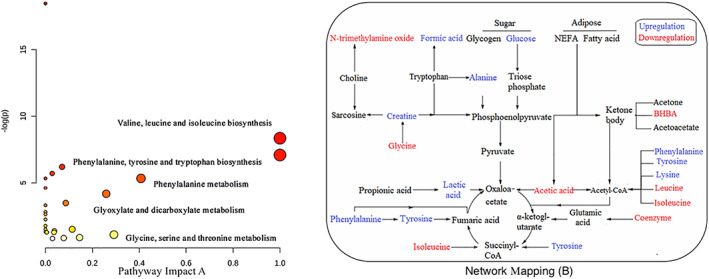
Pathway impact (A) and network mapping (B) of differential metabolites in T2 after administration. A, Bubble plots of the metabolic pathways affected in estrous versus anestrous subjects. The lighter and smaller bubbles represent least affected pathways, whereas the larger and darker bubbles represent the more markedly affected pathways. B, Blue font represents upregulation, red font represents downregulation

The KEGG database was used to analyze the biosynthesis pathway of differentially expressed plasma metabolites in T2 cows before and after treatment (Figure 6A). These included the biosynthesis of valine, leucine and isoleucine, phenylalanine, tyrosine and tryptophan, phenylalanine metabolism, glyoxylic acid and dicarboxylic acid metabolism, and glycine, serine, and threonine metabolism. The 17 metabolites were used to build an interaction network (Figure 6B). Tyrosine, phenylalanine, isoleucine, valine, proline, and lactic acid enter the tricarboxylate acid cycle through the pathways of fumaric acid, succinyl‐CoA, fluorenyl‐ketoglutarate, and oxaloacetate. Tyrosine, phenylalanine, leucine, isoleucine, and lysine enter the tricarboxylic acid cycle or ketone bodies through the acetyl‐CoA pathway. Acetic acid produces acetoacetic acid and amidine‐ketopentan diacid enters the tricarboxylic acid cycle through 2 pathways or generates ketone bodies through acetyl‐CoA. Glucose, alanine, creatine, and glycine all enter the tricarboxylic acid cycle through the pyruvate‐oxaloacetate pathway.

## 4 DISCUSSION

Ketosis is a metabolic disease characterized by elevated levels of ketone bodies (including BHBA), and is classified into clinical ketosis and SCK according to whether there are clinical signs, and whether the BHBA level exceeds 1.20 mm/L.^31^, ^35^ Because there are no obvious clinical signs in SCK, some cows will return to normal on their own and generally, SCK cows are not treated, unless they develop clinical ketosis. Dairy cows with SCK are characterized by oxidative stress and hepatic damage. The ketotic cows usually have abnormal liver function due to hepatitis, fatty liver, or hepatocyte oxidative injury.^36^, ^37^, ^38^ The ketotic cows often have higher AST levels in the serum than healthy cows.^36^, ^37^, ^38^, ^39^, ^40^ AST activity in the serum of >100 IU/L was used as a predictive indicator of dairy cow ketosis.^41^, ^42^ If elevated BHBA and AST levels are not detected, SCK and liver function abnormalities^33^ in the herd will be ignored and not be treated timeously. SCK and liver function abnormalities are potentially harmful to the cow's health and subsequent reproduction.^30^ Some biochemical index such as ALB, ALT, AST, and TP in the plasma might be used to assess the degree of abnormal liver function.^34^, ^43^, ^44^, ^45^ The current study confirmed that CMC‐rGSH alleviated SCK and improved abnormal liver function because plasma levels of BHBA, AST, ALT, and TG decreased gradually, whereas Glu, TC, and HDL increased gradually, and became similar to healthy control cows. This implies that CMC‐rGSH might improve fat metabolism and liver function in SCK cows.

This experiment is an initial exploration of the effect of CMC‐rGSH on SCK, with and without high AST in dairy cows. In the future, a comprehensive study of the effect of CMC‐rGSH on fatty acid metabolism and liver oxidative stress in SCK dairy cows will be conducted in a large‐scale experiment, a positive control must be designed to better evaluate the effect of CMC‐rGSH on SCK cows for improving a lack of the untreated control in this experiment. Although some SCK does spontaneously resolved with time, they would have a longer normal recovery day if there were not any treatment and accompanied by an abnormal liver function according to assessment of serum biochemical indicators including NEFA, BHBA, TG, AST, and liver lipid, etc. Jae^46^ reported 2 regimens to treat SCK cows, 50 SCK cows without any treatment have higher plasma BHBA content (more than 2.0 mmol/L) and lower GLu level (less than 45 md/dL) within 10 days after diagnosis of SCK. Gordon^47^ used butaphosphan, cyanocobalamin and insulin to treat SCK cows, 99 untreated SCK cows had no indication of resolution of SCK 2 weeks after diagnosis, and their serum level of BHBA and GLu were not in the normal range. Caixeta^48^ injected human recombinant FGF21 to improve the liver lipid metabolism of dairy cows showed that the NEFA content in the plasma of the uninjected control cows did not decrease within 8 days, which was significantly higher than that of the treatment group. Zang^49^ added Methyl donor in the feed to alleviate the liver lipid accumulation in dairy cows, the content of TG 46:3 and TG 58:5 in the blood of dairy cows with liver fat deposition in the untreated feed group remained within 14 days, which were significantly higher than the experimental treatment group. In the same way, our experiment shows that serum levels of BHBA, Glu, AST, TG, HDL, LDL, TC in SCK cows with elevated AST returned to normal, of NEFA, ALT were also near normal within 7 days after CMC‐rGSH administration. Therefore, our experimental results showed that CMC‐rGSH effectively shorten the normal recovery day and treat or alleviate SCK in dairy cows, which is consistent with that of previous reports that GSH can improve fatty liver or liver dysfunction in mammals.^20^, ^21^, ^32^

In the current study, metabolomics was used to identify the changes in plasma metabolites, and the metabolic pathways enriched by differential metabolites in T2 cows before and after treatment. One important finding was that the main pathways of plasma glycogenic and ketogenic AAs in SCK cows with high AST were activated after administration of CMC‐rGSH nanoparticles. Plasma levels of glycogenic AAs such as alanine, valine, tyrosine and phenylalanine increased, and ketogenic AAs such as leucine and isoleucine decreased in SCK cows with high AST. Under normal animal metabolism, glutathione is a tripeptide composed of glutamic acid, cystine, and glycine. It is the main prosthetic group of glyceraldehyde dehydrogenase, and a prosthetic group of glyoxalase and triose phosphate dehydrogenase.^50^ It participates in the tricarboxylic acid cycle and sugar metabolism to produce more energy. After exogenous rGSH supplementation, the changes in glycogen and ketogenic AAs confirmed that glutathione activity was enhanced by exogenous rGSH, which successfully balanced the energy supply mode. It has been known that SCK cows had reduced levels of glycogenic AAs and increased ketogenic AAs during early lactation.^51^ Since our metabolomics results showed that after 7 days of administration of rGSH there were lower BHBA and higher GLu in SCK cows with elevated AST by increase in valine, lactate, alanine, lysine, tyrosine, and phenylalanine and decrease in isoleucine and leucine, it suggest that CMC‐rGSH nanoparticles can promote glycogenic AA production, and inhibit the production of ketogenic AAs of in SCK of dairy cows.

There were alterations in other AAs and metabolites in the plasma of SCK cows with high AST after administration of CMC‐rGSH nanoparticles. Lysine is obtained from the diet as a rumen protected nutrient, and is involved in the synthesis and secretion of lipoproteins (such as very low density lipoprotein) to reduce the accumulation of TG in the liver, and alleviate hepatic steatosis.^52^ The metabolomics results indicate that the T2 cows had an increased plasma lysine content after treatment. Plasma oxaloacetic acid content increased due to the increase in lactic acid, alanine, GLu, and creatine since they can enter the tricarboxylic acid cycle to generate oxaloacetic acid.^53^ When SCK occurs, the lipid catabolism increases to produce an increase in acetic acid and BHBA by the acetyl coenzyme A pathway.^54^ In this study, a significant finding was that the plasma levels of acetic acid and BHBA decreased significantly, creatine increased, and glycine decreased after treatment. Lactating dairy cows need a lot of creatine to alleviate NEB through glycine catabolism (as the precursor of creatine),^55^ however the role of the increased creatine, formic acid and lactic acid in SCK of dairy cows after administration of CMC‐rGSH needs to be clarified in future studies. Proline is synthesized from glutamate, but its synthesis is limited, so it is obtained mainly from the diet.^56^ In this study, the plasma proline level decreased after treatment, implying that the degradation of proline increases to meet lactational demand. N‐trimethylamine oxide is an intermediate metabolite of choline and in this study, plasma levels of N‐trimethylamine oxide decreased after treatment, suggesting that choline could be emulsified into cholesterol and released from the liver in the form of phosphatidylcholine.^57^ Choline participates in creatine production through transmethylation, which was consistent with the increase in plasma creatine content.

Another important component of this study was the network mapping of differential plasma metabolites in SCK cows with high AST after administration of CMC‐rGSH nanoparticles. The rGSH is a coenzyme of triose dehydrogenase that participates in the TCA cycle and sugar metabolism in the human and animal, and can activate various enzymes to promote sugar, fat, and protein metabolism.^58^ In addition, rGSH can protect the liver from the damage of oxidative stress because it may directly eliminate free radicals or reduce free radical generation to maintain cell biological functions, and the integrity of cell membranes.^59^, ^60^ According to the interaction network in Figure 5, the increased metabolites (tyrosine, phenylalanine, GLu, alanine, creatine, valine, formic acid, and lactic acid) after administration of CMC‐rGSH in the plasma of SCK cows with abnormal liver function can enter the tricarboxylic acid cycle to provide GLu and energy through the pathways of oxaloacetate, fumaric acid, succinyl‐CoA, and stilbene‐ketoglutarate. Decreased differential metabolites (including acetic acid, leucine, and isoleucine) may reduce the effect of ketogenic AAs via the acetyl‐CoA pathway. Carboxymethyl chitosan‐loaded reduced glutathione may also decrease acetic acid or BHBA in the plasma of SCK cows.

Biochemistry analysis and NMR have therefore confirmed that CMC‐rGSH may improve SCK in dairy cows by some main or other metabolic pathways. During lactation in dairy cows, the intake rate of acetic acid and GLu in the mammary gland is increased for lactose synthesis or energy supply.^61^ During the synthesis of lactose, GLu is first phosphorylated to GLu‐6‐phosphate isomerase (G6P) and then converted to UDP‐galactose. After GLu and UDP‐galactose are taken up by the Golgi apparatus, lactose is synthesized by lactose synthase.^62^ Based on the results of this study, CMC nanoparticles may promote GLu production to provide energy through the pathways of triose phosphate‐phosphoenolpyruvate‐pyruvate‐oxaloacetate‐TCA, or promote GLu uptake and metabolism in the mammary gland through the pathways of GLu‐lactose and GLu‐G6P‐UDP‐galactose‐lactose in dairy cows with SCK and high AST.

## CONFLICT OF INTEREST DECLARATION

Authors declare no conflict of interest.

## OFF‐LABEL ANTIMICROBIAL DECLARATION

Authors declare no off‐label use of antimicrobials.

## INSTITUTIONAL ANIMAL CARE AND USE COMMITTEE (IACUC) OR OTHER APPROVAL DECLARATION

The experiments were managed in accordance with the National Institute of Health Guidelines for the Nursing and Use of Laboratory Animals. All animals were managed in accordance with the standards approved by the Ethics Committee for Animal Welfare and Research of Heilongjiang Bayi Agricultural University China (Number: 20190321‐4).

## HUMAN ETHICS APPROVAL DECLARATION

Authors declare human ethics approval was not needed for this study.

## References

[1] Shaw JC . Studies on ketosis in dairy cattle. VII. The efficacy of B vitamins and methionine in the treatment of ketosis. J Dairy Sci. 1946;29(3):131‐139.

[2] Azagra‐BoroEnjalbert F , Nicot MC , Bayourthe C . Ketone bodies in milk and blood of dairy cows: relationship between concentrations and utilization for detection of subclinical ketosis. J Dairy Sci. 2001;84:1583‐1589. 11286410 10.3168/jds.S0022-0302(01)74511-0

[3] Esposito G , Irons PC , Webb EC , Chapwanya A . Interactions between negative energy balance, metabolic diseases, uterine health and immune response in transition dairy cows. Anim Reprod Sci. 2014;144(3–4):60‐71. 24378117 10.1016/j.anireprosci.2013.11.007

[4] Coleman DN , Vailati‐Riboni M , Elolimy A , et al. Hepatic betaine‐homocysteine methyltransferase and methionine synthase activity and intermediates of the methionine cycle are altered by choline supply during negative energy balance in Holstein cows. J Dairy Sci. 2019;102(9):8305‐8318. 31301838 10.3168/jds.2018-16204

[5] Li Y , Ding HY , Wang XC , et al. An association between the level of oxidative stress and the concentrations of NEFA and BHBA in the serum of ketotic dairy cows. J Anim Physiol Anim Nutr (Berl). 2016;100(5):844‐851. 27079290 10.1111/jpn.12454

[6] Zhang Y , Li X , Zhang H , et al. Non‐esterified fatty acids over‐activate the TLR2/4‐NF‐κb signaling pathway to increase inflammatory cytokine synthesis in neutrophils from Ketotic cows. Cell Physiol Biochem. 2018;48(2):827‐837. 30032133 10.1159/000491913

[7] Berge AC , Vertenten G . A field study to determine the prevalence, dairy herd management systems, and fresh cow clinical conditions associated with ketosis in western European dairy herds. J Dairy Sci. 2014;97(4):2145‐2154. 24534510 10.3168/jds.2013-7163

[8] Baird GD . Primary ketosis in the high‐producing dairy cow: clinical and subclinical disorders, treatment, prevention, and outlook. J Dairy Sci. 1982;65(1):1‐10. 7042782 10.3168/jds.s0022-0302(82)82146-2

[9] Jeong JK , Choi IS , Moon SH , et al. Effect of two treatment protocols for ketosis on the resolution, postpartum health, milk yield, and reproductive outcomes of dairy cows. Theriogenology. 2018;106:53‐59. 29035838 10.1016/j.theriogenology.2017.09.030

[10] Kholif AE . Glycerol use in dairy diets: a systemic review. Anim Nutr. 2019;5(3):209‐216. 31528721 10.1016/j.aninu.2019.06.002 PMC6739259

[11] Zinicola M , Batista CP , Bringhenti L , et al. Effects of recombinant bovine interleukin‐8 (rbIL‐8) treatment on health, metabolism, and lactation performance in Holstein cattle IV: insulin resistance, dry matter intake, and blood parameters. J Dairy Sci. 2019;102(11):10340‐10359. 31495618 10.3168/jds.2019-16337

[12] Gordon JL , LeBlanc SJ , Kelton DF , et al. Randomized clinical field trial on the effects of butaphosphan‐cyanocobalamin and propylene glycol on ketosis resolution and milk production. J Dairy Sci. 2017;100(5):3912‐3921. 28259407 10.3168/jds.2016-11926

[13] Tatone EH , Duffield TF , Capel MB . A randomized controlled trial of dexamethasone as an adjunctive therapy to propylene glycol for treatment of hyperketonemia in postpartum dairy cattle. J Dairy Sci. 2016;11:8991‐9000. 10.3168/jds.2016-11358 27638258

[14] Oikawa S , Elsayed HK , Shibata C , Chisato K , Nakada K . Peripartum metabolic profiles in a Holstein dairy herd with alarm level prevalence of subclinical ketosis detected in early lactation. Can J Vet Res. 2019;83(1):50‐56. 30670902 PMC6318827

[15] Haddad J , Land SC . Redox signaling‐mediated regulation of lipopolysaccharide‐induced proinflammatory cytokine biosynthesis in alveolar epithelial cell. Antioxid Redox Signal. 2002;4(1):179‐193. 11970852 10.1089/152308602753625942

[16] Wu G , Fang YZ , Yang S , Lupton JR , Turner ND . Glutathione metabolism and its implications for health. J Nutrition. 2004;134(3):489‐492. 14988435 10.1093/jn/134.3.489

[17] Chen H , Chen W , Gan LS , Mutlib AE . Metabolism of (S)‐5, 6‐difluoro‐4‐cyclopropylethynyl‐4‐trifluoromethyl‐3,4‐dihydro‐2(1H)‐quinazolinone, a non‐nucleoside reverse transcriptase inhibitor, in human liver microsomes. Metabolic activation and enzyme kinetics. Drug Metab Dispos. 2003;31(1):122‐132. 12485961 10.1124/dmd.31.1.122

[18] Elshorbagy AK , Fredrik JSCL , et al. Exploring the lean phenotype of glutathione‐depleted mice: thiol, amino acid and fatty acid profiles. PLoS One. 2016;11(10):e0163214. 27788147 10.1371/journal.pone.0163214 PMC5082875

[19] Dres D , Mads EB , Maria A , et al. Reduced glutathione as a physiological co‐activator in the activation of peptidylarginine deiminase. Arthritis Res Ther. 2016;18:102. 27149996 10.1186/s13075-016-1000-7 PMC4858833

[20] Zhu G , Zhao C , Han W , et al. Enhanced protection efficacy of reduced glutathione loaded lactose amidated carboxymethyl chitosan nanoparticles on rat's non‐alcoholic fatty liver disease model. J Biomater Tiss Eng. 2018;37:214‐217.

[21] Katarzyna ZK , Anna W , Ewelina U , et al. Distribution of glutathione‐stabilized gold nanoparticles in feline fibrosarcomas and their role as a drug delivery system for doxorubicin—preclinical studies in a murine model. Int J Mol Sci. 2018;19(4):1021. 10.3390/ijms19041021 PMC5979397 29596317

[22] Johnson CH , Ivanisevic J , Siuzdak G . Metabolomics: beyond biomarkers and towards mechanisms. Nat Rev Mol Cell Biol. 2016;17(7):451‐459. 26979502 10.1038/nrm.2016.25 PMC5729912

[23] Griffin JL , Shockcor JP . Metabolic profiles of cancer cells. Nat Rev Cancer. 2004;4(7):551‐561. 15229480 10.1038/nrc1390

[24] Klein MS , Buttchereit N , Miemczyk SP , et al. NMR metabolomic analysis of dairy cows reveals milk glycerophosphocholine to phosphocholine ratio as prognostic biomarker for risk of ketosis. J Proteome Res. 2011;11(2):1373‐1381. 22098372 10.1021/pr201017n

[25] Bertram HC , Yde C , Zhang X , et al. Effect of dietary nitrogen content on the urine metabolite profile of dairy cows assessed by nuclear magnetic resonance (NMR)‐based metabolomics. J Agr Food Chem. 2011;59(23):12499‐12505. 22059599 10.1021/jf204201f

[26] Zhang C , Ping Q , Ding Y . Synthesis and characterization of chitosan derivatives carrying galactose residues. J Appl Polym Sci. 2005;97:2161‐2167.

[27] Daemi H , Barikani M . Synthesis and characterization of calcium alginate nanoparticles, sodium homopolymannuronate salt and its calcium nanoparticles. Scientia Iranica. 2012;19:2023‐2028.

[28] Hongyou Z , Wu L , Xu C , et al. Plasma metabolomic profiling of dairy cows affected with ketosis using gas chromatography/mass spectrometry. BMC Vet Res. 2013;9:186. 24070026 10.1186/1746-6148-9-186 PMC3849279

[29] Li Y , Xu C , Xia C , et al. Plasma metabolic profiling of dairy cows affected with clinical ketosis using LC/MS technology. Vet Quart. 2014;34(3):1‐7. 10.1080/01652176.2014.962116 25299384

[30] Klein MS , Buttchereit N , Miemczyk SP , et al. NMR metabolomic analysis of dairy cows reveals milk glycerophosphocholine to phosphocholine ratio as prognostic biomarker for risk of ketosis. J Proteome Res. 2011;11(2):1373‐1381. 22098372 10.1021/pr201017n

[31] Dale H , Vik‐Mo L , Fjeldheim P . Relationship to energy balance, appetite and ketosis. Nord Vet Med. 1979;31(3):97‐105. 432107

[32] Jiang X , Du B , Zheng J . Glutathione‐mediated biotransformation in the liver modulates nanoparticle transport. Nat Nanotechnol. 2019;14(9):874‐882. 31308501 10.1038/s41565-019-0499-6 PMC7252432

[33] Zhao C , Shu S , Bai YL , Wang D , Xia C , Xu C . Plasma protein comparison between dairy cows with inactive ovaries and estrus. Sci Rep‐UK. 2019;9:13709. 10.1038/s41598-019-49785-8 PMC6757064 31548586

[34] Mostert PF , Bokkers EAM , Middelaar CE , et al. Estimating the economic impact of subclinical ketosis in dairy cattle using a dynamic stochastic simulation model. Animal. 2018;12(1):145‐154. 28637532 10.1017/S1751731117001306

[35] Mezzetti M , Minuti A , Piccioli‐Cappelli F , Amadori M , Bionaz M , Trevisi E . The role of altered immune function during the dry period in promoting the development of subclinical ketosis in early lactation. J Dairy Sci. 2019;102(10):9241‐9258. 31378488 10.3168/jds.2019-16497

[36] Du X , Chen L , Huang D , et al. Elevated apoptosis in the liver of dairy cows with ketosis. Cell Physiol Biochem. 2017;43(2):568‐578. 28934742 10.1159/000480529

[37] Du X , Shen T , Wang H , et al. Adaptations of hepatic lipid metabolism and mitochondria in dairy cows with mild fatty liver. J Dairy Sci. 2018;101(10):9544‐9558. 30100495 10.3168/jds.2018-14546

[38] Taiyu S , Xinwei L , Loor JJ , et al. Hepatic nuclear factor kappa B signaling pathway and NLR family pyrin domain containing 3 inflammasome is over‐activated in ketotic dairy cows. J Dairy Sci. 2019;102(11):10554‐10563. 31495623 10.3168/jds.2019-16706

[39] Kirsten S , Jana F , Ulrich M , et al. Effects of prepartal body condition score and peripartal energy supply of dairy cows on postpartal lipolysis, energy balance and ketogenesis: an animal model to investigate subclinical ketosis. J Dairy Res. 2014;81(3):257‐266. 24594287 10.1017/S0022029914000107

[40] Felix DG , Rodrigo M , Víctor P , et al. Relationship among blood indicators of lipomobilization and hepatic function during early lactation in high‐yielding dairy cows. J Vet Sci. 2011;2(3):251‐255. 10.4142/jvs.2011.12.3.251 PMC3165154 21897097

[41] Yu C , Jiang Z , Wei Y , et al. Predictive value of plasma parameters in the risk of postpartum ketosis in dairy cows. J Vet Res. 2017;61:91‐95. 29978059 10.1515/jvetres-2017-0011 PMC5894404

[42] Yuhang S , Bo W , Shi S , et al. Critical thresholds of liver function parameters for ketosis prediction in dairy cows using receiver operating characteristic (ROC) analysis. Vet Quart. 2015;9:37‐41. 10.1080/01652176.2015.1028657 25831953

[43] Zhang J , Wang G , Zhao C , et al. ^1^H‐NMR plasma metabolomic profiling of ovarian quiescence in energy balanced postpartum dairy cows. Vet Quart. 2018;38:1‐10. 10.1080/01652176.2018.1473660 PMC6830969 29733756

[44] Knight JA . Liver function tests: their role in the diagnosis of hepatobiliary diseases. J Infus Nurs. 2005;28(2):108‐117. 15785331 10.1097/00129804-200503000-00004

[45] Bobe G , Young JW , Beitz DC . Invited review: pathology, etiology, prevention, and treatment of fatty liver in dairy cows. J Dairy Sci. 2004;87(10):3105‐3124. 15377589 10.3168/jds.S0022-0302(04)73446-3

[46] Jae KJ , In‐Soo C , Sung‐Ho M , et al. Effect of two treatment protocols for ketosis on the resolution, postpartum health, milk yield, and reproductive outcomes of dairy cows. Theriogenology. 2018;106:53‐59. 29035838 10.1016/j.theriogenology.2017.09.030

[47] Gordon JL , Duffield TF , Herdt TH , Kelton DF , Neuder L , LeBlanc SJ . Effects of a combination butaphosphan and cyanocobalamin product and insulin on ketosis resolution and milk production. J Dairy Sci. 2017;100(4):2954‐2966. 28215889 10.3168/jds.2016-11925

[48] Caixeta LS , Giesy SL , Krumm CS , Perfield JW 2nd , Butterfield A , Boisclair YR . Fibroblast growth factor‐21 (FGF21) administration to early‐lactating dairy cows. II. Pharmacokinetics, whole‐animal performance, and lipid metabolism. J Dairy Sci. 2019;102(12):11597‐11608. 31548064 10.3168/jds.2019-16696

[49] Zang Y , Saed S , Myers WA , et al. Methyl donor supplementation suppresses the progression of liver lipid accumulation while modifying the plasma triacylglycerol lipidome in periparturient Holstein dairy cows. J Dairy Sci. 2018;102:11597‐11608. 10.3168/jds.2018-14727 30471914

[50] Forman HJ , Zhang H , Rinna A . Glutathione: overview of its protective roles, measurement, and biosynthesis. Mol Aspects Med. 2009;30(1–2):1‐12. 18796312 10.1016/j.mam.2008.08.006 PMC2696075

[51] Marczuk J , Brodzki P , Brodzki A , Kurek Ł . The concentration of free amino acids in blood serum of dairy cows with primary ketosis. Pol J Vet Sci. 2018;21(1):149‐156. 29624009 10.24425/119033

[52] Lee C , Lobos NE , Weiss WP . Effects of supplementing rumen‐protected lysine and methionine during prepartum and postpartum periods on performance of dairy cows. J Dairy Sci. 2019;102(12):11026‐11039. 31548066 10.3168/jds.2019-17125

[53] Gualdrón‐Duarte LB , Allen MS . Increased anaplerosis of the tricarboxylic acid cycle decreased meal size and energy intake of cows in the postpartum period. J Dairy Sci. 2017;100(6):4425‐4434. 28342606 10.3168/jds.2016-12104

[54] Kessel S , Stroehl M , Meyer H , et al. Individual variability in physiological adaptation to metabolic stress during early lactation in dairy cows kept under equal conditions. J Anim Sci. 2008;86(11):2903‐2012. 18599666 10.2527/jas.2008-1016

[55] Megahed AA , Hiew MWH , Ragland D , Constable PD . Changes in skeletal muscle thickness and echogenicity and plasma creatinine concentration as indicators of protein and intramuscular fat mobilization in periparturient dairy cows. J Dairy Sci. 2019;102(6):5550‐5565. 30954258 10.3168/jds.2018-15063

[56] Wu G , Davis PK , Flynn NE , Knabe DA , Davidson JT . Endogenous synthesis of arginine plays an important role in maintaining arginine homeostasis in postweaning growing pigs. J Nutrition. 1997;127(12):2342‐2349. 9405584 10.1093/jn/127.12.2342

[57] Xu G , Ye J , Liu J , et al. Effect of rumen‐protected choline addition on milk performance and blood metabolic parameters in transition dairy cows. Asian Austral J Anim Sci. 2006;19(3):390‐395.

[58] Gangwar C , Saxena A , Patel A . Effect of reduced glutathione supplementation on cryopreservation induced sperm cryoinjuries in Murrah bull semen. Anim Reprod Sci. 2018;192:171‐178. 29559193 10.1016/j.anireprosci.2018.03.005

[59] Franco R , Schoneveld OJ , Pappa A , Panayiotidis MI . The central role of glutathione in the pathophysiology of human diseases. Arch Physiol Biochem. 2007;113(4‐5):234‐258. 18158646 10.1080/13813450701661198

[60] Wu JH , Batist G . Glutathione and glutathione analogues; therapeutic potentials. Biochim Biophys Acta. 2013;1830(5):3350‐3353. 23201199 10.1016/j.bbagen.2012.11.016

[61] Azagra‐Boronat I , Tres A , Massot‐Cladera M , Franch À , Castell M , et al. Lactobacillus fermentum CECT5716 supplementation in rats during pregnancy and lactation affects breast milk composition. J Dairy Sci. 2020;103(4):1982‐1992. 32008776 10.3168/jds.2019-17384

[62] Rigout S , Lemosquet S , Van Eys JE , et al. Duodenal glucose increases glucose fluxes and lactose synthesis in grass silage‐fed dairy cows. J Dairy Sci. 2002;85(3):595‐606. 11949864 10.3168/jds.S0022-0302(02)74113-1

